# Effect of ligninolytic axenic and coculture white-rot fungi on rice straw chemical composition and in vitro fermentation characteristics

**DOI:** 10.1038/s41598-022-05107-z

**Published:** 2022-01-21

**Authors:** Osmond Datsomor, Zhao Gou-qi, Lin Miao

**Affiliations:** College of Animal Science and Technology, Yangzhou University, Yangzhou, 225009 China

**Keywords:** Biological techniques, Microbiology

## Abstract

The study sought to investigate the potentials of axenic cultures of *Pleurotus ostreatus*, *Phanerochaete chrysosporium* and their coculture (*P. chrysosporium* and *P. ostreatus*) to break down lignin and to enhance the rumen fermentability of rice straw. Rice straw was fermented by two lignin-degrading fungi, namely, *P. ostreatus*, *P*. *chrysosporium* and its coculture (*P*. *ostreatus* and *P*. *chrysosporium*) with uninoculated straw as control under solid-state fermentation employing a completely randomized research design. The coculture exhibited a mutual intermingling plus inhibition interaction. The fungi treatment increased the crude protein from (5.1%) in the control to (6.5%, 6.6%, and 6.7%) in the *P*. *ostreatus*, *P*. *chrysosporium* and coculture respectively. The coculture treated straw had a lower lignin content (5.3%) compared to the *P*. *chrysosporium* (6.2%) with the *P*. * ostreatus* recording the least (3.3%) lignin fraction. Treatment of rice straw with coculture improved the in vitro dry matter digestibility (68.1%), total volatile fatty acids (35.3 mM), and total gas (57.4 ml/200 mg) compared to *P*. * chrysosporium* (45.1%, 32.2 mM, 44.4 ml/200 mg) but was second to *P*. * ostreatus* (75.3%, 38.3 mM, 65.6 ml/200 mg). Instead of an anticipated synergistic effect from the coculture, a competitive antagonistic effect was rather observed at the end of the study, a condition that can be attributed to the coculture behavior.

## Introduction

Rice straw is an abundant and inexpensive energy-rich source from crop residue. Therefore, it is used as a part of the ration for ruminants in most rice-cultivating countries^1^ particularly during the dry season when fresh forage is limited. Rice straw contains 25–45% cellulose, 25–30% hemicellulose, and 10–15% lignin^2^. However, its utilization is limited due to its high lignin and low protein content^3^. Therefore, lignin removal coupled with protein enrichment of straw is a vital pretreatment strategy. Physical, chemical, and/ biological delignification treatments have been established to increase the nutritive value, nutrient digestibility, and utilization of rice straw.

Biological pretreatment is the most preferable because it is practical, safe, and environmentally friendly^4^. White-rot fungi continues to receive increased research for lignocellulosic biomass delignification as they are the only organism known to effectively and efficiently mineralize lignin into water and carbon dioxide^5^. The fungal mycelium also adds protein to the substrate. Several white-rot fungi, including *P*. *ostreatus* and *P*. *chrysosporium*, which have been screened under axenic culture conditions, have exhibited high ligninolytic potential^4^. However, fungi, as they normally grow near each other, establish a wide spectrum of interactions^6,7^ with the abiotic factors in the environment and the substrate. Therefore, depending on the kind of substrate used and the association established, diverse results^6,8,9^ can be expected.

Some species can form synergistic interactions^10^ by enzyme action^11^ significantly improving the degradation of lignocellulose materials. Therefore, it has been hypothesized that a coculture may result in a comparatively higher delignification of straw material resulting in an improved in vitro fermentation index compared to monocultures. For instance, Chen et al.^12^ reported higher degradation ratio of lignin (26.38%) and cellulose (33.29%) using a *P*. *chrysosporium* and *T*. *viride* coculture. Tobacco stalk substrate treated with *T*. *hirsuta* S13 and *P*. *ostreatus* S18 coculture also achieved a twofold lignin degradation rate^13^. Recently, Kaur et al.^14^ reported a maximum laccase (Lac), lignin peroxidase (LiP), and manganese peroxidase (MnP) activities of 2.40 nkat/gds, 37.92 nkat/gds, and 62.50 nkat/gds respectively using *P*. * ostreatus* and *P*. * chrysosporium* coculture on rice straw. These independent studies demonstrate the potential of white-rot fungal consortia to alter the cell wall component of various straws through their enzymatic activities.

These independent studies however did not either ascertain the type of interaction or the in vitro feed evaluation assay such as digestibility, volatile fatty acids and in vitro gas production which is a critical step in in vitro and in vivo feed evaluation. Therefore, in this study, axenic cultures of white-rot fungi; *P*. *ostreatus*, *P*. *chrysosporium*, and their coculture were employed in the delignification of rice straw. The study also sought to establish the nature of the interaction, the effect of the axenic and coculture on the chemical components, in vitro dry matter digestibility, volatile fatty acids, and gas production. This would provide useful information on ration formulation strategies using white-rot treated rice straw for ruminant nutrition during the dry season.

## Materials and methods

### Fungi

Two of the ligninolytic white-rot fungi, *P*. *chrysosporium* CGMCC 3.7212 and *P*. *ostreatus* CGMCC 3.7292 were used for the study. They were provided by China Agricultural University in Beijing, China. They were grown on a malt extract agar (MEA) plate culture-medium (malt extract 20 g; agar 20 g; nutritional yeast 2 g; per L) and stored at 4 °C. Agar plates were prepared using an autoclaved sterilized MEA (malt extract 20 g; agar 20 g; nutritional yeast 2 g; per L; 121 °C to 25 min), inoculated with a 0.5 cm^2^ piece of the fungi and incubated at 25 ± 1 °C until mycelium covered the entire surface of the plates.

### Paired interaction on agar plates

Interspecies interactions of the two fungi species were investigated by placing square inoculum plugs of the same size at two opposite poles of the MEA plates 40 mm apart. Interactions between opposing mycelia were assessed visually every other day using the protocol of Rayner and Boddy^15^.

### Millet spawn preparation

Millet grains were washed in water and boiled for 15 min. The boiled grains were transferred onto a sieve to drain. The grains were packed into two polyethylene mycobag (25 cm wide and 37 cm height) until it was three-quarters full and then autoclaved at 121 °C for 45 min. The content of each mycobag was permitted to cool to room temperature and then separately inoculated aseptically with five 1 cm^2^ of mycelium agar and sealed. The contents of the polyethylene mycobags were shaken manually to ensure uniform mixing of the mycelium with the grains. It was then incubated in a ventilated incubator at 25 ± 1 °C until the mycelia colonized all the grains. The spawns were then removed, allowed to cool and stored in a cold room at 4 °C to stop the mycelia from further growth and for future use.

### Substrate preparation and inoculation

The rice straw used in the present study was collected from the rice field of Yangzhou University, Jiangsu, China. Rice straw was chopped into 2–3 cm lengths and packed into a 2 mm net mesh. The packed meshed rice straw was placed in a barrel of water thrice the weight of the straw and left overnight for the water to penetrate the inner structures of the straw. The soaked rice straw was hung in the open air using a hanger to allow the excess water to drain. 250 g of the wet substrate was weighed into sixteen (16) polyethylene mycobags and sterilized in an autoclave at a temperature of 121 °C for 1 h. The autoclaved mycobags were cooled to room temperature and each straw mycobag was inoculated with millet spawn at 5% (w/w) of straw. The control was prepared the same as the treatment groups except the spawn inoculum. The inoculated mycobags were shaken to ensure uniform spawn distribution and incubated at a temperature of 25 ± 1 °C and 75–80% humidity. The mycobags with fungal spawn were cultured for 30 days according to the guideline of Oei^16^. After incubation, the mycobags with treated substrate including mycelium was oven-dried at 64 °C for 48 h. The dried fungi treated rice straw were ground over a 1 mm sieve using a miller machine (CM100, Beijing Yongguangming Co., Ltd., China) to obtain a homogenous sample and stored for further chemical and in vitro analysis.

### Experimental design

A single factor completely randomized design was used to assign treatments to experimental units. The control group consisted of autoclaved uninoculated rice straw. The experimental group was rice straw treated with the axenic culture of *P*. *ostreatus*, *P*. *chrysosporium* and their coculture. All of the experiments were performed in quadruplicate.

### Chemical composition analysis

The samples were analyzed for dry matter by drying them at 105 °C in an oven dryer (DHG-9123A, Zhengzhou Wollen Instrument Equipment Co., Ltd., Shanghai, China) for 3 h. Nitrogen (N) contents were analyzed using the Kjeldahl method according to the AOAC^17^, and crude protein determined by multiplying the nitrogen (N) by 6.25. The neutral detergent fiber (NDF), acid detergent fiber (ADF), cellulose, hemicellulose and acid detergent lignin (ADL) analysis was performed as described by Van Soest et al.^18^ employing an Ankom 2000 automated fiber analyzer (ANKOM Technology, Mecedon, New York, USA). Samples (0.5–1 g) were placed into polyester mesh bags (Ankom F57, ANKOM Technology, Mecedon, New York, USA) and sealed. Bags and 2000 ml of neutral detergent were put into the automatic fiber analyzer at 100 °C for 60 min. Then, the bags were washed to neutral with distilled water, dried and weighed. The dried residue was represented as NDF. The remaining samples and 2000 ml of acid detergent were put into the automatic fiber analyzer at 100 °C for 60 min. Then, the bags were washed to neutral with distilled water, dried and weighed. The dried residue was represented as ADF. The content of hemicellulose was calculated as the difference between NDF and ADF. The dried residue was soaked in 72% (v/v) H_2_SO_4_ and kept at 25 °C for 2 h. After that, the bags were washed to neutral with distilled water, dried and weighed. The remaining samples were kept at 550 °C for 3 h in a tared crucible and reweighed to calculate ADL loss. The content of cellulose was calculated as the difference between ADF and ADL. Ash content was determined by carbonization of the samples in a muffle furnace (1200 Kiln, Jinan Cyeeyo Instruments Co., Ltd., China) at 550 °C for at least 3 h. Organic matter (OM) was calculated as the difference between DM and ash content. All calculations were on a dry matter basis expressed in percentages.

### In vitro fermentation

The maintenance of the rumen-fistulated Holstein cow, and procedure of rumen fluid collection were approved by the Animal Care Committee of Yangzhou University (Jiangsu, China). Fresh rumen fluid was collected from three rumen-fistulated Holstein cow fed corn silage and oat straw-based diet. The fluid was filtered through four lays of cheesecloth and was mixed in a 1:2 (v/v) ratio buffer solution (Buffer A: 13.2 g CaCl_2_·2H_2_O, 10.0 g MnCl_2_·4H_2_O, 1.0 g CoCl_2_·6H2O and 8.0 g FeCl_3_·6H_2_O per 100 ml; Buffer B: 35.0 g NaHCO_3_ and 4.0 g NH_4_HCO_3_ per 1000 ml; Buffer C: 5.7 g Na_2_HPO_4_ and 0.6 g MgSO_4_·7H_2_O and 6.2 g KH_2_PO_4_ per 1000 ml) under continuous flushing with CO_2_ according to the procedure of Menke et al.^19^. 200 mg of oven-dried control group and experimental group samples were weighed into a 100 ml glass vial. Each glass vial received 30 ml of buffered rumen fluid and were incubated in an incubator shaker (Model THZ—320, Jinghong Devices, Shanghai, China) at 39 °C for 48 h along with blanks. All the groups (control, experimental, and blank) were conducted in quadruplicate.

### In vitro gas, volatile fatty acids and dry matter digestibility

The head-space gas pressure in each glass vial was recorded at 0, 3, 6, 12, 24, 36 and 48 h using a digital pressure transducer gauge (Model DPG1000B15PSIG-5, Cecomp Electronics, Libertyville, IL, USA) fitted with a 22-gauge hypodermic needle following the procedure of Theodorou et al.^20^. The gas production values were corrected for the blank incubation (gas of sample − the gas of blank). The volume of gas was determined using the mathematical equation: *Vgas* = *Vj* × *Pps*i × 0.068004084, where *Vgas* is the gas volume at 39 °C, ml, *Vj* is the vial volume headspace of liquid, ml, *Ppsi* is the pressure of the vial, psi.

At the end of 48 h of incubation, the vials were taken out of the incubator and placed into an ice-water bath to stop fermentation. The vials were then uncapped; fermentation mixture pH was measured using a pH meter (Model PHS-3C, Puchun Co., Ltd., Shanghai, China) and contents were transferred into conical centrifuge tubes (50 ml). The conical centrifuge tubes were then centrifuged using Eppendorf centrifuge 5810R (Fisher Scientific Co, USA) at 8000×*g* and 4 °C for 15 min to obtain a supernatant and non-fermented solid residue. Each sample’s supernatant was transferred into centrifuge tubes (1.5 ml) after which 1 ml supernatant was mixed with 0.2 ml 20% metaphosphoric acid (containing 60 mM crotonic acid), and stored overnight at 4 °C to be later used for volatile fatty acids (VFA) concentration determination. The VFA concentration was determined using a gas chromatography–mass spectrometer (GC–MS 9800, Shanghai Kechuang Chromatographic Instrument Co., Ltd., Shanghai, China) equipped with a thermal conductivity detector Agilent capillary column (30 m × 0.32 mm × 0.25 μm, DB-FFAP: TDX-01). The temperature of the injector, column and detector was 200 °C, 110 °C and 200 °C respectively. The carrier gas was nitrogen, with a 50 ml/min flow rate and 1 µI injection volume. The non-fermentable solid residues of each sample were dried at 65 °C overnight and weighed. The in vitro dry matter digestibility (IVDMD) was estimated as the difference in weight between the dried non-fermentable solid residues and the initial weight of the conical centrifuge tube (50 ml). Blank corrections were conducted for IVDMD.

### Statistical analysis

A completely randomized research design was used to evaluate the effects of axenic culture of white-rot fungal species and its coculture on the chemical composition, in vitro pH, VFA, gas production of control, and experimental group samples. Data were analyzed as a single factor Analysis of variance (ANOVA) using SPSS, version 21.0 (IBM Corp., Armonk, NY, USA). Post-hoc multiple comparisons with Duncan’s significant test at a significance level of 0.05 was performed to determine the significance between experimental groups. Prior to conduction the ANOVA, the assumption of homogeneity of variances was tested and satisfied based on Levene’s test (*P* > 0.05).

## Results

### Interactions between *P. ostreatus* and *P. chrysosporium* fungal isolates in dual culture

**Figure 1:**
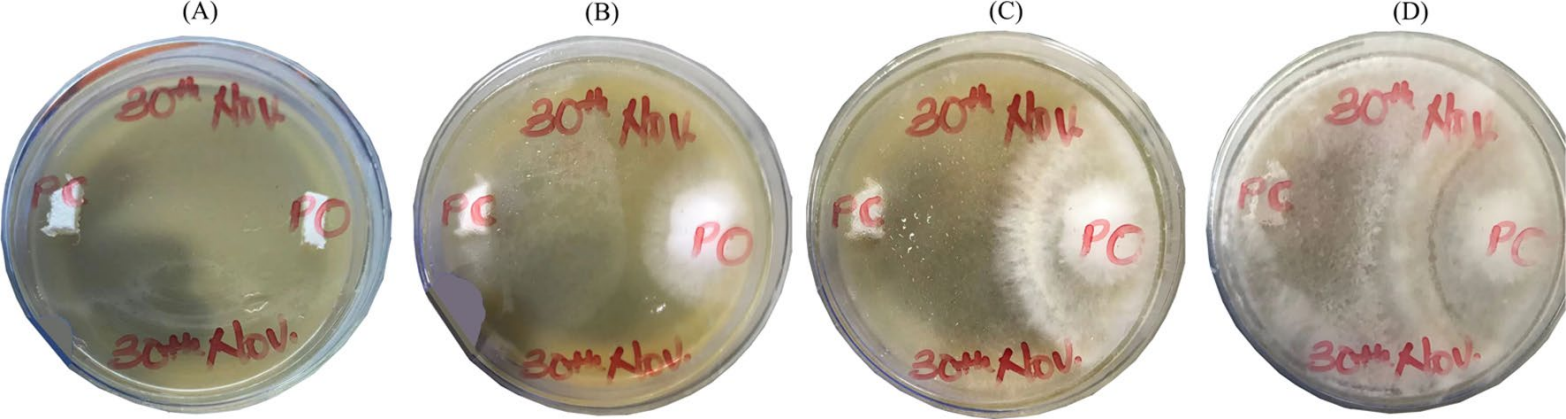
Mycelial confrontation test between P. chrysosporium and P. ostreatus in MEA solid plate medium: (**A**) 1 day, (**B**) 3 days, (**C**) 5 days (**D**) 7 days.

**Figure 2:**
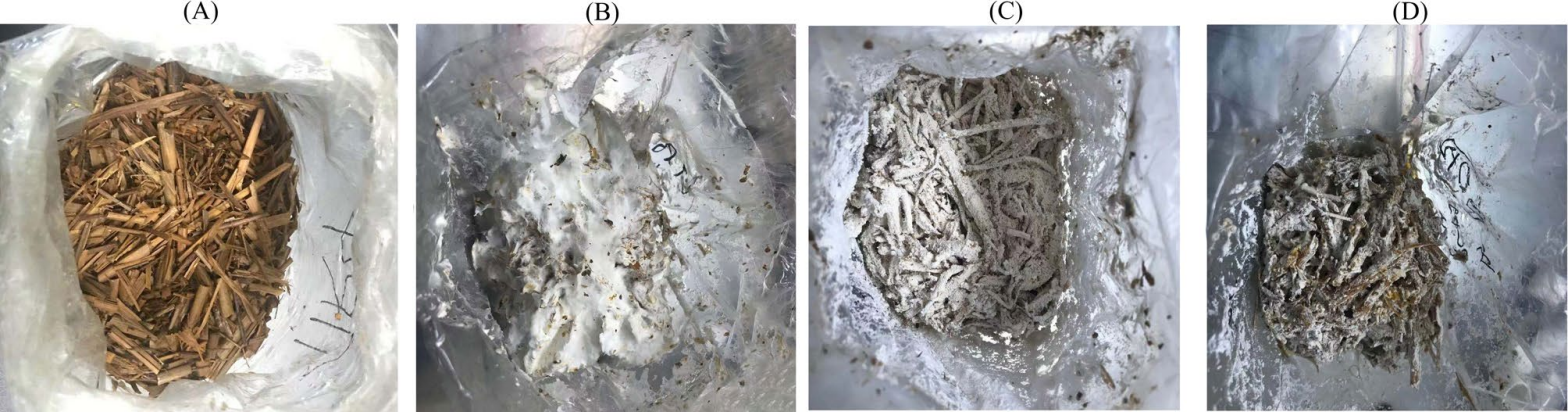
Rice straw after 30 days incubation: (**B**) untreated, (**B**) P. ostreatus, (**C**) P. chrysosporium, (**D**) Coculture.

To determine the type of interaction that occurred between the *P*. *ostreatus* and the *P*. *chrysosporium*, a visual mycelium confrontation test was carried out. After plating (Fig. 1A), *P*. *chrysosporium* was observed to grow faster than *P*. *ostreatus* (Fig. 1B). *P*. *chrysosporium* and *P*. *ostreatus* mycelia made an initial contact leading to the formation of a distinct dense whitish barrage (Fig. 1C). The whitish barrage formed was evident of a mutual intermingling owing to cytoplasmic contact of the fungi isolates via hyphal fusion and subsequent increased continual fusion of mycelia mass (anastomosis). A brownish to yellow colouration of the barrage was observed. The growth of barrage mycelia towards *P*. *ostreatus* and *P*. *chrysosporium* was restricted, resulting in the formation of a partial inhibition region (Fig. 1D). The axenic white-rot fungi species and coculture all thrived well on rice straw with no mold visibly detected (Fig. 2A–D).

### Chemical composition of rice straw treated with *P. ostreatus* axenic fungi, *P*. *chrysosporium* axenic culture and coculture

Treatment of the rice straw with *P*. *ostreatus*, *P*. *chrysosporium* and its coculture had a positive effect on the substrate’s chemical composition and cell wall contents (Table 1). Except the hemicellulose, significant differences (*P* < 0.05) were recorded between the treatments for all the parameters (DM, OM, Ash, NDF, ADF, ADL and Cellulose) that were assessed compared to the control with the *P*. *ostreatus* performing considerably better compared to the *P*. *chrysosporium* and the coculture. Similarly, the performance of the coculture was superior to *P*. *chrysosporium*. Although the treatments positively impacted the crude protein fraction compared to the control, there was no significant difference (*P* > 0.05) among the treatments.

**Table 1:** Chemical composition (%) of rice straw after incubation with axenic and coculture white-rot fungi.

Parameters	Control	*P. ostreatus*	*P*. *chrysosporium*	Coculture
DM	94.25 ± 0.20^a^	88.48 ± 0.36^b^	81.58 ± 0.36^d^	84.73 ± 0.27^c^
OM	81.23 ± 0.16^a^	73.26 ± 0.40^b^	62.81 ± 0.43^d^	68.81 ± 0.42^c^
CP	5.07 ± 0.15^b^	6.50 ± 0.06^a^	6.57 ± 0.24^a^	6.69 ± 0.05^a^
Ash	13.02 ± 0.06^d^	15.21 ± 0.11^c^	18.77 ± 0.07^a^	15.93 ± 0.20^b^
NDF	70.63 ± 0.24^a^	61.00 ± 0.21^b^	54.44 ± 0.08^d^	57.12 ± 0.29^c^
ADF	51.82 ± 0.02^a^	45.46 ± 0.11^b^	39.39 ± 0.06^d^	42.27 ± 0.06^c^
ADL	8.16 ± 0.01^a^	3.27 ± 0.07^d^	6.18 ± 0.03^b^	5.26 ± 0.05^c^
Cellulose	43.66 ± 0.02^a^	42.19 ± 0.05^b^	33.21 ± 0.07^d^	37.00 ± 0.09^c^
Hemicellulose	18.81 ± 0.26^a^	15.54 ± 0.10^b^	15.08 ± 0.10^b^	14.90 ± 0.26^b^

All values are mean ± standard error.

^a–d^Values with different superscript letters in a row represent significant difference (*P* < 0.05).

### pH, IVDMD and volatile fatty acids profile of rice straw treated with *P*. *ostreatus* axenic fungi, *P*. *chrysosporium* axenic culture and coculture

Except for the pH, the rice straw treated with *P*. *ostreatus*, *P*. *chrysosporium* and coculture compared to the control had a considerable impact on all the parameters; IVDMD, total VFA, and the individual VFA assessed in this study (Table 2). The IVDMD and the total VFA concerning *P*. *ostreatus* were markedly higher than both the control and the other treatments. Even though the coculture produced considerably higher total VFA and IVDMD than the control and *P*. *chrysosporium*, the total VFA and IVDMD produced by the *P*. *chrysosporium* was markedly inferior to the control. The *P*. *chrysosporium*, on the other hand, did yield the highest amount of branched short-chain fatty acids; isobutyric, isovaleric and valeric. However, the amount of valeric acid produced with respect to *P*. *ostreatus*, the coculture and the control were comparable. On the other hand, the coculture did yield a substantially higher amounts of isovaleric and isobutyric compared to the *P*. *ostreatus*. The differences that were recorded in the production of acetic acid had *P*. *chrysosporium* recording the least proportion while *P*. *ostreatus* was higher among the treatments. However, the acetic acid produced by *P. ostreatus* was not comparable to that of the control. The performance of the *P*. *ostreatus* with respect to the A:P was preferred to the control as well as the other treatments; coculture and the *P*. chrysosporium. Similarly, *P*. *ostreatus* produced the highest amount of propionic acid markedly while the *P*. *chrysosporium* recorded the least amount.

**Table 2:** pH, IVDMD and VFA from rice straw after incubation with axenic and coculture white-rot fungi.

Parameter	Control	*P*. *ostreatus*	*P*. *chrysosporium*	Coculture
pH	6.84 ± 0.08	6.85 ± 0.04	6.97 ± 0.08	6.69 ± 0.06
IVDMD (%)	51.02 ± 0.61^c^	75.34 ± 0.41^a^	45.09 ± 0.50^d^	68.08 ± 0.35^b^
Total VFA (mM)	34.38 ± 0.10^c^	38.31 ± 0.07^a^	32.24 ± 0.08^d^	35.27 ± 0.06^b^
**Individual VFA (%total VFA)**				
Acetic acid	67.06 ± 0.02^a^	66.13 ± 0.04^b^	64.21 ± 0.12^d^	65.03 ± 0.02^c^
Propionic acid	22.04 ± 0.02^c^	25.16 ± 0.08^a^	20.19 ± 0.12^d^	23.04 ± 0.02^b^
A:P	3.09 ± 0.01^b^	2.63 ± 0.01^d^	3.18 ± 0.01^a^	2.83 ± 0.01^c^
Isobutyric acid	1.08 ± 0.04^d^	2.50 ± 0.01^c^	4.88 ± 0.17^a^	3.09 ± 0.04^b^
Butyric acid	6.02 ± 0.01^a^	5.34 ± 0.07^b^	4.21 ± 0.10^d^	5.09 ± 0.02^c^
Isovaleric acid	3.09 ± 0.08^c^	1.09 ± 0.04^d^	4.21 ± 0.21^a^	3.14 ± 0.09^b^
Valeric acid	1.13 ± 0.04^b^	0.52 ± 0.03^b^	2.43 ± 0.64^a^	1.05 ± 0.02^b^

All values are mean ± standard error. A:P, acetate propionate ratio.

^a–d^Values with different superscripts in a row represent significant different (*P* < 0.05).

### In vitro gas volume of rice straw treated with *P. ostreatus* axenic fungi, *P. chrysosporium* axenic culture and coculture

A significant difference (*P* < 0.05) was recorded between the various treatments with respect to the control during the incubation period (Table 3). The volume of gas produced at the various time interval by *P*. *ostreatus* was considerably higher than that of both the control and the other treatments; *P*. *chrysosporium* and coculture. On the other hand, the gas production from the *P*. *chrysosporium* treated rice straw during the period was markedly the lowest among all the treatments. Nonetheless, that of the control was not comparable to the coculture.

**Table 3:** In vitro gas volume from rice straw after incubation with axenic and coculture white-rot fungi.

Gas volume (ml/200 mg)	Control	*P*. *ostreatus*	*P*. *chrysosporium*	Coculture
Gv 3 h	3.33 ± 0.01^c^	4.62 ± 0.01^a^	2.34 ± 0.02^d^	4.27 ± 0.01^b^
Gv 6 h	13.07 ± 0.01^c^	15.61 ± 0.07^a^	10.55 ± 0.01^d^	13.42 ± 0.04^b^
Gv 12 h	23.32 ± 0.01^c^	28.07 ± 0.01^a^	20.11 ± 0.01^d^	25.32 ± 0.01^b^
Gv 24 h	42.26 ± 0.02^c^	47.59 ± 0.01^a^	37.92 ± 0.03^d^	44.38 ± 0.01^b^
Gv 36 h	49.06 ± 0.18^c^	63.25 ± 0.02^a^	43.08 ± 0.18^d^	54.80 ± 0.01^b^
Gv 48 h	51.41 ± 0.04^c^	65.60 ± 0.75^a^	44.39 ± 0.02^d^	57.40 ± 0.18^b^

All values are mean ± standard error. Gv, Gas volume.

^a–d^Values with different superscript letters in a row represent significant difference (*P* < 0.05).

## Discussion

The observed interaction with respect to the behavior of the coculture in the study is consistent with the report by Windram et al.^21^ who reasoned that when mutual intermingling coexists with inhibition, an antagonistic reaction can occur because the barrage reaction requires cytoplasmic contact, and this may form abnormal and even lethal fusions between the mycelia. The coloration of interacting mycelia fronts is attributed to the melanin biosynthesis and improved phenoloxidase activity at the tips of the hyphae^10^. The brownish coloration according to Hammel and Cullen^22^ could be an indication of free radicals present at the tips of the hyphal region which invigorate lignin and lignin-type polymer decomposition. Furthermore, the vigorous growth of fungi on the substrate which is a precondition for the fermentation process ensured the rapid growth of the mycelia on the substrate and thus, inhibited the unwanted microorganisms (molds or bacteria) from contaminating the substrate^23^.

With the exception of ash and crude protein, the observed general decline in chemical composition of the treated rice straw compared to the control was unavoidable because the fungi required the nutrients present in the substrate for their self-proliferation. In the present study, *P*. *chrysosporium* treated rice straw recorded the least DM which is similar to Zheng et al.^24^. This was due to the relatively fast growth of *P*. *chrysosporium* compared to the coculture and *P*. *ostreatus* treatments^25^. Similarly, *P*. *chrysosporium*, *P*. *ostreatus* and the coculture-treated rice straw also recorded a substantial decline in the organic matter with recorded losses of 23%, 10% and 15%, respectively. This trend is similar to the report by Kerem et al.^25^ who, after 28 days of treating cotton stalk with *P*. *chrysosporium* and *P*. *ostreatus* observed a 55% loss and 20% loss of organic matter respectively although losses were much higher than observed in the present study. The decline in organic matter indicates successful colonization and utilization of biomass macromolecules by the fungi. Fungi treated rice straw had a positive effect on the protein content, which is consistent with the studies of Tuyen et al.^26^. The increased protein content originates from the breakdown of the organic material, which is utilized by the fungi for its growth and development^27^. *P*. *chrysosporium* treated rice straw of all the treatments recorded the highest percentage of ash while the *P*. *ostreatus* recorded the least amount of ash. A notable increase in the ash content of the fungi-treated rice straw compared to the control was observed. The increase in ash was due to the degradation and subsequent release of minerals within substrate by the fungi^28^.

*Phanerochaete chrysosporium*, *Pleurotus ostreatus* and coculture fungi substantially degraded the fibrous fraction of the rice straw after 30 days of incubation. This demonstrates explicitly that the white-rot fungi as biological pretreatment are experts in degrading the structural carbohydrate and lignin in lignocellulosic tissues deriving organic carbon for their energy demands. This is in agreement with the report of Rahman et al.^29^, who stated that fungi-treated straw contained lower NDF, ADF, ADL, hemicellulose and cellulose content than untreated straw. In the current study, *P*. *chrysosporium* treated rice straw had lower cellulose but higher lignin content. This agrees with the considerably higher lignin and lower cellulose in biodegraded wheat straw recorded by Tuyen et al.^26^. According to Salvachúa et al.^30^ proteomic assay of the secretome of *P*. *chrysosporium* revealed several diverse glycoside hydrolases (GHs) made up of a cascade of enzymes involved in the absolute breakdown of cellulose. *P*. chrysosporium which is considered a non-selective/simultaneous delignifier^31^ consumes large amount of the cellulose with small loss in lignin or shows no preference to lignocellulosic. This results in higher degradation of the available cellulose while lignin degradation decelerates. *P*. *ostreatus*, on the other hand, produces diverse ligninolytic enzymes that exclusively attack lignin over cellulose^32^ and is thus termed a selective white-rot fungus as the decomposition of lignin is associated with marginal loss in cellulose^33^.

However, the microbial consortium in the current study failed to significantly outperform both axenic cultures in achieving a highly delignified and holocellulose rich biomass. According to Reiss et al.^34^, it could be due to microbes not sharing enzymatic pathways/activities, thus the synergy to promote the division of labour among its members but instead required the maximum potential of a single strain. Mechanisms to protect the energy resource and defend the habitat might have thus occurred in the microbial consortium. This suggests the inferior performance of *P*. *chrysosporium* compared to control and the other treatments, while the *P*. *ostreatus* did perform comparatively better than the coculture. This can be attributed to the variation in the cultural behavior and condition^35^.

The pH is a vital index that reflects the rumen environment. In the present study, the axenic and coculture treatment of rice straw did not affect the rumen pH, and this agrees with the finding of Khonkhaeng and Cherdthong^36^. The values in the current study were all within the usual range (> 6.3) for optimal rumen metabolism^37^. Removal of lignin is directly associated with an enhancement in in vitro digestibility. The comparatively reduced IVDMD of *P*. *chrysosporium* treatment in spite of the substantial reduction in lignin compared to the untreated straw could be attributed to the comparatively high depletion in the DM and cellulose. Although the *P*. *chrysosporium* had an excellent effect on the ADL as the degradation exposed the holocellulose, it did not record a positive effect on the nutritional value of the substrate because there was substantial simultaneous degradation of the exposed cellulose. The resultant adverse effect on the amount of cellulose accessible to the rumen microorganism considerably led to a further decline in substrate digestibility compared to untreated rice straw. Similarly, treatment of naked oat with *P*. *chrysosporium*, according to Zheng et al.^24^ led to a further decline in IVDMD than the untreated straw. The superior performance of *P*. *ostreatus* compared to control, and coculture can be attributed to the ability of *P*. *ostreatus* to selectively degrade lignin than cellulose in lignocellulosic biomass.

Despite the slight decline in the cellulose level of the *P*. *ostreatus* treated rice straw, it did record a resultant higher effect on the ADL degradation and IVDMD compared to the control and the coculture. This result is similar to the improved dry matter digestibility previously reported by Atalar and Çetİnkaya^38^. This is because the degradation of the lignin was not accompanied by excessive cellulose loss. This suggests that rumen microorganisms had access to enough cellulose for hydrolysis. Even though van Kuijk et al.^39^ had expressed that the extent to which each fungus contributes to coculture is not always clear and thus leaves the possibility that one culture is left, the improvement in the IVDMD of the coculture in this study compared to the *P*. *chrysosporium* and the untreated rice straw could partly be attributed to the contribution of the *P ostreatus* in the coculture. This observation is similar to the increased in vitro digestibility achieved on spruce wood degradation via a *P*. *chrysosporium* and *P*. *ostreatus* coculture^40^.

Volatile fatty acid (VFAs), last product after the fermentation of carbohydrate serves as the energy reserve for ruminants and reflects the digestibility of feed. The total VFA in various fungi treatment groups aligned with the observed IVDMD and gas production. The observed increase in the total VFA of the *P*. *ostreatus* treated rice straw while the use of *P*. *chrysosporium* resulted in a decline is coherent with the findings of Niu et al.^41^. This is because, the substrate with higher digestibility value implies more access to fermentable carbohydrates by rumen microbes, which in turn yields higher total VFA compared to substrate with lower digestibility value. The coculture had the ability to increase the total VFA of rice straw but not to the level achieved by *P*. *ostreatus* treated rice straw. This could be attributed to the decline in lignin which reduced the cell wall recalcitrance to an extent allowing cell wall constituent’s hydrolysis. The value of a feed is denoted by its total VFA yield along with its molar proportion, particularly the A:P fraction with a rise in feed efficiency in the rumen linked with a lower A:P ratio. In the present study, *P*. *ostreatus* treated rice straw recorded the least A:P ratio (2.63) which is similar to the observation (3.27) made by Zuo et al.^42^ only that his value was comparatively higher. Just as in the present study, *P*. *chrysosporium* treated rice straw, recorded the worse A:P of 3.18, it was similar to a worse A:P of 3.55 reported by Niu et al.^41^ using *P*. *chrysosporium* in a similar study. The observed variation as earlier mentioned could be attributed to effects of the culture behavior and/or difference in the culturing conditions such as the type of substrate and incubation period among others.

In vitro gas production results from the direct fermentation of feedstuff and indirectly from the buffering of short chain fatty acids (SCFA). The volume of gas produced from rumen microbial fermentation of feedstuffs in vitro is positively related to its digestibility^43^. Much as the output from the current study recorded a progressive increase in the volume of gas produced in all the substrates, the volume of gas produced was comparatively higher in rice straw treated with *P*. *ostreatus*. This observation suggests an improvement in the digestibility of the fungi-treated substrate, which can be ascribed to the decrease in the fiber components due to selective degradation of lignin over cellulose. This observation agrees with the increase in the total volume of gas production (IVGP) reported by Tuyena et al.^44^. Although the total IVGP of the *P*. *chrysosporium* treated rice straw increased, it was the least among all the treatments in the current study. This is similar to the comparative decline in total in vitro gas production from wheat straw treated with *P*. *chrysosporium*^25^. This is because *P*. *chrysosporium* extensively consumed cell wall polysaccharides, resulting in a lower IVDMD. The IVGP of coculture being superior to control and *P*. *chrysosporium* but lower than *P*. *ostreatus* treated straw is clear evidence of antagonistic action originating from the axenic culture species combination.

## Conclusion

An evaluation of the interaction between the *P. chrysosporium* and the *P. ostreatus* showed a prevailing mutual intermingling plus inhibition relationship, although the species could grow together on the same medium.

The results as a whole suggest that the effect of the coculture on the substrate resulted in a competitive antagonistic instead of an anticipated synergistic effect.

The use of *P. ostreatus*, in the pretreatment of rice straw for dry season feeding of ruminant is preferred considering its ability to effectively and efficiently degrade lignin and enhance the nutritional value of the material. This is based on its effect on the improved in vitro dry matter digestibility, volatile fatty acids and gas production compared to the control and other treatment; *P. chrysosporium* and *P. ostreatus*–*P. chrysosporium* coculture.

## Data Availability

The datasets generated and analyzed are available from the corresponding author on reasonable request.
